# A Mouldy Stomach

**Published:** 1900-08-11

**Authors:** 


### A MOULDY STOMACH.

Dr. Max Einhorn,[1] of New York, has been making observations on the occurrence of mould in the human stomach. That the mould fungus does sometimes infest the stomach is well known, but the extent to which its presence does harm seems as yet not to have been ascertained with any approach to accuracy. Its persistent presence must, however, be regarded as abnormal, for although isolated fungi may exist for a short time in the stomach without any detriment, they do not find in the normal organ favourable soil for further development. Under normal conditions they become intimately mixed with the chyme, and are carried onward, living or dead, through the pylorus. Thus to find entire colonies which are microscopically perceptible suggests something wrong in the stomach; for any such considerable growth would be possible only if a colony of the fungi had infested a fold of the surface of the gastric mucous membrane, and had obtained such a hold upon it as to resist the onward passage of the chyme and those muscular movements by which that onward flow is caused. Granting, then, that the presence of such an amount of mould in the stomach as to show itself by visible particles coming away in the wash-water after stomach lavage is to be taken as indicating the existence of a morbid state, the further question arises whether the fungus itself is responsible for the malady with which it may be associated, and this is the question which still remains unanswered. Dr. Einhorn says that he has met with mould-formation, particularly in two groups of gastric affections: first, in cases of intense hyperchlorhydria, occasionally attended with hypersecretion and vomiting; and second, in gastralgia with normal or reduced gastric secretion. It cannot, he says, be denied that in many of these cases the mould flakes become smaller in number, and disappear after treatment by lavage, &c., and that in connection with this a subjective improvement is observed in the condition of the patient. Yet it cannot be said with certainty that the mould produced the pathological process in the stomach, for we find cases analogous in every respect without the presence of mould fungi. That is as far as we can go at present, except that, so far as the fungus is concerned, the treatment would seem to be the use of irrigation of the stomach in the fasting state, following which a spray of nitrate of silver solution appears occasionally to be of some utility.

[1] Med. Rec., June 16.

